# Definition, conservation and epigenetics of housekeeping and tissue-enriched genes

**DOI:** 10.1186/1471-2164-10-269

**Published:** 2009-06-17

**Authors:** Xinwei She, Carol A Rohl, John C Castle, Amit V Kulkarni, Jason M Johnson, Ronghua Chen

**Affiliations:** 1 Rosetta Inpharmatics LLC, a wholly owned subsidiary of Merck and Co., Inc., 401 Terry Avenue North, Seattle, WA 98109, USA

## Abstract

**Background:**

Housekeeping genes (HKG) are constitutively expressed in all tissues while tissue-enriched genes (TEG) are expressed at a much higher level in a single tissue type than in others. HKGs serve as valuable experimental controls in gene and protein expression experiments, while TEGs tend to represent distinct physiological processes and are frequently candidates for biomarkers or drug targets. The genomic features of these two groups of genes expressed in opposing patterns may shed light on the mechanisms by which cells maintain basic and tissue-specific functions.

**Results:**

Here, we generate gene expression profiles of 42 normal human tissues on custom high-density microarrays to systematically identify 1,522 HKGs and 975 TEGs and compile a small subset of 20 housekeeping genes which are highly expressed in all tissues with lower variance than many commonly used HKGs. Cross-species comparison shows that both the functions and expression patterns of HKGs are conserved. TEGs are enriched with respect to both segmental duplication and copy number variation, while no such enrichment is observed for HKGs, suggesting the high expression of HKGs are not due to high copy numbers. Analysis of genomic and epigenetic features of HKGs and TEGs reveals that the high expression of HKGs across different tissues is associated with decreased nucleosome occupancy at the transcription start site as indicated by enhanced DNase hypersensitivity. Additionally, we systematically and quantitatively demonstrated that the CpG islands' enrichment in HKGs transcription start sites (TSS) and their depletion in TEGs TSS. Histone methylation patterns differ significantly between HKGs and TEGs, suggesting that methylation contributes to the differential expression patterns as well.

**Conclusion:**

We have compiled a set of high quality HKGs that should provide higher and more consistent expression when used as references in laboratory experiments than currently used HKGs. The comparison of genomic features between HKGs and TEGs shows that HKGs are more conserved than TEGs in terms of functions, expression pattern and polymorphisms. In addition, our results identify chromatin structure and epigenetic features of HKGs and TEGs that are likely to play an important role in regulating their strikingly different expression patterns.

## Background

The expression of most genes varies between different cell and tissue types and between different development and physiological states. Some genes, however, are constitutively expressed in all tissues and their expression levels are comparatively constant across different cell types. These genes have been referred to as housekeeping genes (HKGs) and are hypothesized to constitute a small set of genes required to maintain minimum basic cellular function [1]. In contrast to the expression pattern of HKGs, tissue enriched genes (TEG) are highly expressed in one particular tissue type and are either not expressed or are expressed at much lower levels in other tissues. TEGs are generally responsible for the specialized functions of the particular tissues or cell types in which they are expressed and can therefore serve as biomarkers of specific biological processes or tissues. Since many diseases involve tissue- or organ-specific processes, TEGs may also be good candidate drug targets. HKGs, in contrast, have been widely used as experimental controls and normalization references for gene transcription and expression experiments, including RT-PCR, qPCR, Western blotting and microarray studies. The expression of many of the genes currently used for such purposes, however, varies across different cell types and conditions, and consequently there is a need for a better set of HKGs that have stable, high expression levels across a large number of tissues.

The genomic organization of HKGs is comparatively compact: intronic regions, coding regions and the intergenic spaces are shorter for HKGs than for other genes [2,3], and HKGs are strongly clustered in the human genome [4], suggesting selection for economy in transcription and translation [3] and genomic co-regulation of broadly expressed genes. HKGs, as a result of their critical role in basic cell maintenance, are subject to stronger purifying selection and therefore evolve more slowly than TEGs in terms of sequence mutation [5]. It is less well understood to what extent the functions and expression patterns of HKGs are conserved across species, whether HKGs are conserved at the genomic structure level and how polymorphic HKGs and TEGs are among different individuals within a species. To address these questions, we sought to define a high quality set of HKGs and then analyze the conservation of HKGs in terms of functions and expression patterns. We also analyzed the distribution of genomic component, such as segmental duplication, copy number variation regions and ultra conserved elements, which are closely related to conservation.

The regulatory mechanisms underlying the differential expression patterns of HKGs compared to TEGs are also poorly characterized. Chromatin structure and epigenetic modifications of genomic structure have been documented to regulate gene expression and affect replication, recombination and DNA repair [6,7] through various mechanisms including nucleosome positioning and occupancy, histone modification (mainly acetylation and methylation) and DNA cytosine methylation [8,9]. Abnormal changes in chromatin structure have been linked to disease, particularly cancer [10]. Investigation of the differences in chromatin structure and epigenetic modification between HKGs and TEGs, consequently, may provide insight into epigenetic contributions to transcriptional patterns and the mechanisms of gene regulation and disease.

Here we use microarray gene expression profiling and analysis to compile a set of 1,522 high quality HKGs that are highly expressed in 42 normal tissues and show minimal fluctuations in expression level across these tissues. Similarly, we describe the identification of 975 TEGs. These genes from both categories are potentially useful laboratory experimental controls. The distinct expression patterns of HKGs and TEGs and the high quality of these sets also provide an opportunity to enhance our understanding of transcriptional and epigenetic regulatory mechanisms. We compare and contrast the genomic and epigenetic properties of HKGs and TEGs, and identify epigenetic factors that may contribute to the underlying mechanisms of expression regulation differences between HKGs and TEGs.

## Results and Discussion

### HKGs and TEGs

We identified 1,522 HKGs from a total of 18,149 genes in 42 normal human tissues monitored on the microarray (see Methods). This list of HKGs was used for analysis of genomic and epigenetic features (see Additional file 1). We also identified 975 TEGs from a subset of 29 representative tissues. These TEGs are expressed at much higher level in one single tissue than any other tissues (see Methods. Additional file 2). TEGs were found in 26 tissues, while no TEGs meeting our criteria were identified in spleen, colon and CD4+ T-cells.

**Figure 1:**
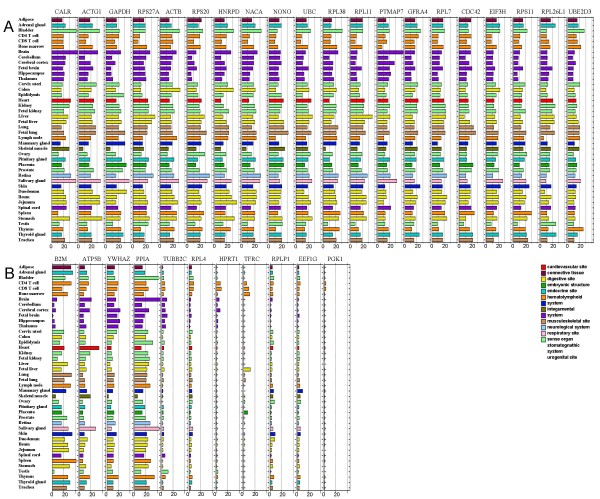
**Expression of housekeeping genes across normal tissues**. (A) Expression levels of the top 20 housekeeping genes, as ranked by average expression intensity, are shown. Three commonly used housekeeping genes, *GAPDH*, *ACTB *and *UBC*, are among this list. (B) Expression patterns for commonly used or commercially available housekeeping genes that do not meet the criteria defined here for housekeeping genes are shown. The length of the horizontal bars represents the expression intensity of the genes in microarray.

The 20 HKGs with the highest and most consistent expression (See Methods) were selected from this list as the best candidates to serve as reference HKGs in laboratory experiments (Figure 1A). Three of the 20 highest quality HKGs, *GAPDH*, *ACTB *and *UBC*, are commonly used HKGs for experimental controls. Expression data for several other commonly used or commercially available HKGs ([11] and ) not included in the top 20 HKG lists (Figure 1B) illustrate that genes commonly used as controls do not necessarily show high expression with low variance across diverse tissues, and in fact, the expression of some of these genes varies by more than an order of magnitude across tissues.

There have been other recent efforts to identify HKGs in human tissues to serve as experimental references or internal controls [12-16], but these studies have significant shortcomings. One study surveyed a much smaller set of 7012 potential candidate genes [12], while another one used a much smaller set of 15 tissues [16]. Others were based on microarray data from heterogeneous sources lacking systematic experimental controls [15] or were based on *in silico *predictions [14]. The HKGs identified here are based on high quality microarray expression data systematically gathered from a large and diverse set of tissues. This is a systematically and experimentally defined set of HKG which have both high expression and low fluctuation across all major organ/tissues.

The human and mouse transcriptome in multiple tissues have been surveyed in microarray studies [17-19] that built foundations for studies on housekeeping and tissue-specific genes [13,19,20]. Large collections of EST and SAGE data have also been used to identify HKG [21] and tissue-specific genes [16]. Comparing HKGs of this study and other studies based on microarray [13] or EST datasets [21], significant portions of genes in the three HKG sets overlap, while the HKG list described here has the fewest genes unique in a single study, suggesting our fluctuation-controlled microarray approach is more conservative than the other methods that either depend on sampling or representation in an EST dataset [21] or lack control of variation across different tissues [13] (see Additional file 3). We also compared our TEGs in testis, prostate, liver and skin with tissue-specific genes from another study [19] (see Additional file 4). A significant portion of our TEGs overlap with this human tissue-specific gene study, particularly for liver, in which 70% of the defined TEGs are identical. Other tissues are more discrepant, as a result of different tissue selection for the surveys and different criteria used to identify these genes.

**Figure 2:**
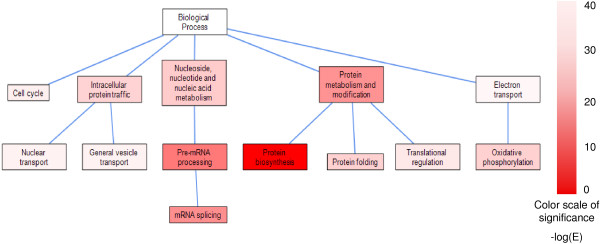
**Biological processes enriched in housekeeping genes**. Enriched biological processes (E value < 0.05) and their ancestors from the Panther Ontology are shown in a hierarchical structure. E values were calculated by hypergeometric distribution with a Bonferroni correction. The intensity of color is proportional to the significance of gene enrichment (-log(E value), ranging from the lightest 2.3 to the darkest 39.1).

We next investigated the functions of HKGs by testing for enrichment of Panther Biological Process annotations [22]. As expected, HKGs are enriched for biological processes related to basic maintenance of the cell, including protein biosynthesis, pre-mRNA processing, cell cycle and intracellular protein trafficking (Figure 2). In contrast, TEGs were observed to be enriched largely for tissue-specific biological processes, as expected (see Additional file 5). For instance, TEGs of bone marrow, brain, kidney, liver and skeletal muscle are enriched in immunity and defense, synaptic transmission, ion transport, lipid metabolism and muscle contraction, respectively. These biological processes are presumably less likely to be essential for cell maintenance and survival.

### Conservation of functions and expression patterns in HKGs across species

The enriched biological processes observed for HKGs suggest that a minimum set of functions are required for cells to survive, but it is not obvious to what extent these functions and genes will be conserved across other mammalian and eukaryotic species. To address this question, we used the number of orthologs of human genes in other eukaryotic species as identified by NCBI HomoloGene [23] as an indication of functional conservation across species (Table 1). In general, fewer orthologs can be identified as the evolutionary distance between human and the target species increases. Human HKGs, however, are significantly more likely to have orthologs in other species relative to other genes (p = 0.01), while TEGs are less likely to have orthologs (p = 0.065). This difference is particularly striking in invertebrates, where the ratios between the fraction of HKGs and TEGs with orthologs in fly, worm or yeast are 3.9 (54%:14%), 3.8 (46%:12%) and 11.5 (23%:2%), respectively. This analysis suggests that human HKG functions, mostly involving gene expression, are functionally conserved in model organisms such as worm and yeast, while many human TEG-related functions were acquired after the divergence between humans and lower organisms.

**Table 1:** Cross-species conservation of housekeeping genes and tissue enriched genes

	HKG orthologs		TEG orthologs		Orthologs of all genes on array	
	
	Count	Ratio	Count	Ratio	Count	Ratio
Human	1,380	100%	843	100%	17,641	100%
Mouse	1,352	98%	799	95%	16,614	94%
Rat	1,258	91%	763	91%	15,312	87%
Dog	1,287	93%	697	83%	15,127	86%
Fly	752	54%	122	14%	5,111	29%
Worm	639	46%	103	12%	3,973	23%
Yeast	324	23%	20	2%	1,591	9%

P value	0.010		0.065		N/A	

The ratio indicates the fraction of human genes with orthologs in other species. 1380, 843 and 17,641 of total HKGs, TEGs and all genes on the array, respectively, are represented in HomoloGene and these numbers were used to calculate the ratio of orthologs.

HKGs were identified in this study on the basis of their observed expression in human tissues. To determine if this expression pattern is conserved across species, we examined the expression of human HKGs orthologs in mouse, rat, and dog gene expression profiling data sets (unpublished data) for these species (Table 2). Relative to orthologs for all genes on the microarray, HKG orthologs are more likely to remain highly expressed with small variations in other mammalian species. The average intensity level and CV (coefficient of variance) of the HKG orthologs are both comparable to their counterparts in human, suggesting that the expression pattern of HKGs is conserved among mammalian species and those HKGs in human are likely to be HKGs in other mammals. This finding is also supported by a recent study in which universally expressed genes across tissues were found generally more ancient in origin compared with specifically expressed genes [24].

**Table 2:** Expression of human housekeeping genes in mammals

	Average Intensity		CV of Intensity	
	
	All Genes in array	HKG Orthologs	All Genes in Array	HKG Orthologs
Human	1.08	5.18	2.27	0.45
Mouse	1.18	4.92	3.90	0.54
Rat	1.33	3.94	3.49	0.57
Dog	1.62	5.48	3.16	0.48

### Distribution of segmental duplication, copy number variation sites and ultraconserved elements in HKGs and TEGs

It is estimated more than 5% of the human genome is composed of segmental duplications (SDs), duplicated genomic blocks ranging from 1 kb to over 200 kb [25]. To test if the high expression of HKGs is related to higher copy numbers in the genome, we calculated the distribution of SDs [26] and copy number variations (CNVs) [27] in HKGs and TEGs (See Methods. Table 3). To our surprise, SDs are not enriched in HKGs, but are enriched two-fold in TEGs relative to RefSeq genes. Generally, the copy numbers of genes are positively correlated with gene expression [28-30]. Our results clearly show that the high expression level of HKGs does not rely on the redundancy of gene copies. CNV sites are only slightly enriched in HKGs, but are strongly enriched in TEGs. The increased polymorphism of TEGs is concordant with their weaker selection constraints than HKGs, and probably resulted in by the enriched SDs which are predisposed to non-allelic homologous recombination, chromosomal rearrangement and copy number variations [31].

**Table 3:** Genes with segmental duplication, copy number variation and ultraconserved elements

	Segmental Duplication			Copy Number Variation			Ultra Conserved Elements			Total number of genes
	
	Number of genes	Percent of all genes	P-value	Number of genes	Percent of all genes	P-value	Number of genes	Percent of all genes	P-value	
HKGs	88	5.8%	>0.05	218	14.3%	0.03	35	2.3%	0.03	1,522
TEGs	119	12.2%	2.9E-11	176	18.1%	7.6E-05	8	0.8%	0.28	975
RefSeq genes	1195	6.6%	N/A	2528	13.9%	N/A	156	0.9%	N/A	18149

We also calculated the distribution of ultra conserved elements (UCEs) which are sequences that are absolutely conserved (100% identical) between orthologous regions of the human, rat, and mouse genomes [32]. Consistent with the slower evolution of HKGs, UCEs are significantly enriched in HKGs and not changed in TEG (Table 3). HKGs have been found to evolve more slowly than TEGs at the sequence level point mutation [5]. The distribution of SD, CNV and UCE demonstrated that HKGs are also more conserved than TEGs with respect to genomic structural changes.

### Enriched CpG islands at HKG transcription start sites

It has long been known that HKGs are associated with CpG islands [33]. Recently it was found that the lack of CpG islands around the transcription start site is associated with a higher degree of tissue specificity [34]. These studies, however, lacked a systematic representation of HKGs or TEGs, either testing only a limited number of experimentally confirmed HKGs or relying on the redundancy of ESTs as an indirect indicator of HKGs. We compared the CpG island distribution (data from ) 500 bp around transcription start sites (TSS) and end sites in HKGs and TEGs using the systematic HKG and TEG gene lists assembled here (Table 4). Both the fraction of genes containing CpG islands and the density of CpG islands at TSS are correlated with the expression patterns with TEGs being depleted for CpG islands and HKGs showing enrichment of CpG islands relative to RefSeq genes in general. A recent EST based study showed HKGs primarily use CpG-dependant core promoters [24]. However, our report is a systematic and quantitative demonstration of enrichment of CpG islands at HKG TSS and depletion at TEG TSS. We also observed that at transcription end sites, the occurrence of CpG islands significantly decreases for all groups of genes, and both HKGs and TEGs show a slight depletion of CpG islands (Table 4). Despite the fact that the sequence of HKGs is generally more conserved than other genes, another analysis showed that HKGs tend to have reduced upstream sequence conservation particularly within CpG rich genes [20]. Enrichment of CpG islands in the TSS of HKGs may play a role in this reduced upstream conservation.

**Table 4:** CpG islands at transcription start and end sites

	Transcription start sites			Transcription end sites			
		
	Number of genes with CpG islands (ratio)	CpG density (/bp)	P-value	Number of genes with CpG islands (ratio)	CpG density (/bp)	P-value	Total number of genes in class
HKGs	1,230 (80.8%)	0.563	3.5E-40	84 (5.5%)	0.02	3.0E-14	1,522
TEGs	279 (28.6%)	0.155	3.4E-40	49 (5.0%)	0.02	6.6E-13	975
RefSeq genes	8,881 (48.9%)	0.321		2086 (11.5%)	0.053		18,149

### Chromatin structure and epigenetic modifications in HKGs

We next examined differences in chromatin structure and epigenetic modifications, including nucleosome occupancy, histone modifications, and DNA methylation between HKG and TEGs as possible mechanisms contributing to the differential expression patterns of these two groups of genes.

**Figure 3:**
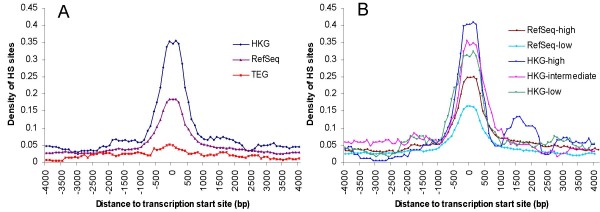
**DNase I hypersensitive (HS) site enrichment at transcription start sites of housekeeping genes**. (A) The average density of HS sites detected in CD4+ T cells [35] is shown for each 500 bp sliding window advancing 100 bp each time within 4 kb of the transcription start site for three gene groups, HKGs, all RefSeq genes, and TEGs. (B) RefSeq genes and HKGs are further partitioned into subgroups based on their expression level (probe intensity) in CD4+ T cell profiling microarray: RefSeq-low (intensity < 1), RefSeq-high (intensity > 1), HKG-low (1< intensity < 3), HKG-intermediate (3 < intensity < 6), HKG-high (intensity > 6). The surge of density of HS site around 1500 bp in the HKG-high group is likely an artifact of the small sample size in this group.

DNase I hypersensitive (HS) sites, formed by nucleosome-free chromatin regions, have been used to identify locations of many different types of regulatory regions including enhancers, silencers, promoters, insulators, and locus control regions [35,36]. Figure 3A shows the distribution of HS sites in CD4+ T cells [35] at TSS for HKGs, TEGs and RefSeq genes. The density of HS sites peaks at TSS, as expected, and rapidly drops to background levels beyond 1 kb from TSS. HKGs show an elevated density of HS sites relative to RefSeq genes, indicating that TSS of HKGs are less likely to be packaged into nucleosomes and are more exposed to transcription and regulatory factors. HS site density in TEGs is low, consistent with the low levels of expression of these genes in CD4+ T cells, the cell type from which the HS sites data were generated.

The HKGs identified in this study are highly expressed in CD4+ T cells (and all other tissues), leading to the possibility that the differences observed in HS site density seen for HKGs and TEGs may reflect only the overall expression level of these genes in CD4+ T cells, rather than the difference in expression patterns across tissues. To address this question, we partitioned both the HKGs and RefSeq genes into subgroups based on their expression level in CD4+ T cells: low, intermediate and high (Figure 3B). While a correlation between HS site density and expression level is still observed across the subgroups for either HKGs or RefSeq genes, the HKG-low expression subgroup (average expression intensity: 2.55) has a higher HS site density than the RefSeq-high expression subgroup (average intensity: 3.48), clearly demonstrating that the HS site density is not simply a function of gene expression level in CD4+ T cells, but also correlates with the high levels of expression across different tissues. A recent study showed the positive association of CpG density with the distribution of HS sites across different tissues [37], suggesting that the increase in HS sites in HKGs may be related to high CpG density. Another possible explanation for this observation is that HKGs may contain sequence elements at their TSS that inhibit formation of nucleosomes, leading to high promoter accessibility and higher expression levels of these genes across different tissues. Further investigation of TSS sequences and more HS site mapping in other tissues would be necessary to test this hypothesis.

**Figure 4:**
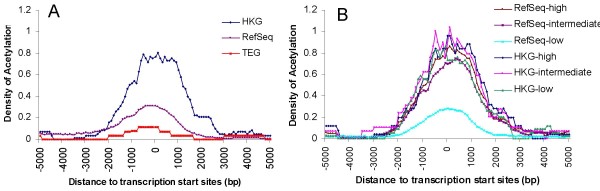
**Histone acetylation enrichment at transcription start sites of housekeeping genes**. (A) Each dot represents the percentage of sites with histone acetylation [39] in a 500 bp sliding window advancing 100 bp each time within 5 kb of the transcription start site. (B) HKGs and RefSeq genes from panel A are further partitioned into subgroups based on their expression level (probe intensity) in Hep G2 cells: RefSeq-low (intensity < 1), RefSeq-intermediate (1 < intensity < 3), RefSeq-high (intensity >3), HKG-low (1 < intensity < 3), HKG-intermediate (3 < intensity < 6), HKG-high (intensity > 6).

Histone acetylation regulates gene expression by allowing transcription factors access to promoters in the chromatin by neutralizing the positive charge of histone tails and weakening their contact with negatively charged DNA [38]. Using histone acetylation data of chromosome 21 and 22 derived from liver Hep G2 cells[39], we show that the acetylation ratio of genes peaks at the TSS (Figure 4). The histone acetylation density observed for HKGs is higher than RefSeq genes on average, while the density observed for TEGs, which are not expressed in liver Hep G2 cells, is almost at the background level (Figure 4A). This observation is consistent with recent genome-wide studies in which histone acetylation is positively correlated with transcription factor binding or gene expression [40-42]. To study the correlation of gene expression with histone acetylation, HKGs and RefSeq genes were partitioned into subgroups based on their expression level in Hep G2 cells. 74% of all RefSeq genes have low expression (intensity < 1) in the Hep G2 cells and the higher levels of histone acetylation levels for HKGs relative to RefSeq genes was maintained. The difference in histone acetylation between RefSeq genes and HKGs with medium (1 < intensity < 3) and high (intensity > 3) expression levels, however, virtually disappears. It appears that histone acetylation density depends only on gene expression level, indicating that histone acetylation of TSS does not contribute to the expression pattern of HKGs across tissues (Figure 4B). Interestingly, while histone acetylation density is low for genes with low expression levels, it is essentially identical for all subgroups with expression intensity >1. This observation of a threshold-like relationship suggests that histone acetylation of TSS may be a necessary condition for gene expression and may serve as a "transcriptional switch" that opens the chromatin structure and allows other transcription factors to regulate gene expression. Significant differences between HKGs and TEGs in both nucleosome occupancy and histone acetylation suggest that the regulation of gene expression for these different groups is affected by multiple epigenetic factors.

**Figure 5:**
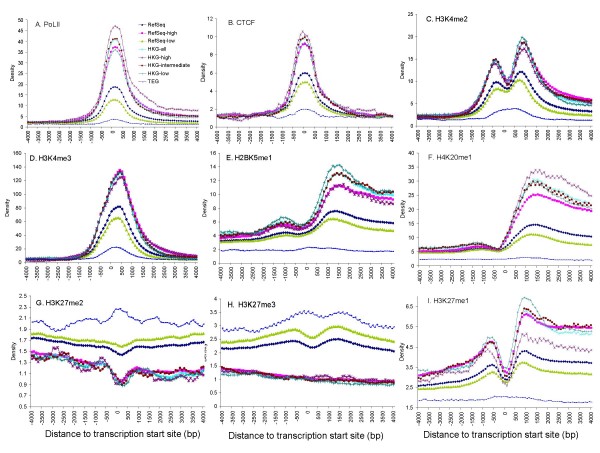
**Density of histone methylation and transcription factor binding sites at transcription start sites for HKGs, TEGs, and RefSeq genes**. The density of transcription factor binding or histone methylation of different sites in CD4+ T-cells [43] is shown for a 500 bp sliding window advancing 100 bp each time near transcription start sites for HKGs, RefSeq genes, TEGs and their subgroups, based on expression levels. Gene subgroups are as defined in Figure 3. The densities of transcription factor binding or histone methylation of genes are displayed as follows: (A) Pol II, (B) CTCF, (C) H3K4me2, (D) H3K4me3, (E) h2BK5me1, (F) H4K20me1, (G) H3K27me2, (H) H3K27me3, (I) H3K27me1.

We also compared the transcription factor binding and histone methylation density between HKGs and TEGs using data collected in CD4+ T cells [43] (Figure 5). As expected, binding of the transcription factor pol II and regulatory factor CTCF reaches a peak level at the exact position of TSS. CTCF is a zinc finger protein with diverse regulatory functions, including a role in mediating chromatin interactions to form the genomic three-dimensional structure[44]. Recent studies on HKG clusters around the α-Globin and β-Globin loci suggest chromatin loop/hub forms during the transcription of the gene clusters [45,46]. Our results suggest CTCF plays a role in HKG transcription. Transcription factor binding is highest for HKGs among all gene groups, while transcription factor binding of TEGs, which are not expressed in CD4+ T cells, is near background levels (Figure 5A, B). The patterns observed for different histone methylation sites of HKGs and TEGs are complex, likely reflecting the complex relationship between histone methylation and transcription (Figure 5C–I). Differences in the shape of the distribution are observed between HKGs, TEGs and RefSeq genes for some histone methylations. When similar distribution shapes are observed, histone methylations may be either positively or negatively correlated with the expression level. These features suggest that histone methylation likely contributes to the differential gene expression patterns of these genes in a complex fashion.

DNA methylation in mammals occurs on cytosine residues of CpG dinucleotides, which may lead to formation of heterochromatin, imprinting and transcriptional repression [47]. The distribution of genome-wide DNA methylation [48] in HKGs, RefSeq genes and TEGs (see Additional file 6) shows that DNA methylation peaks at TSS for all gene groups and that there is no significant difference of DNA methylation levels between HKGs, TEGs and RefSeq genes in either sperm cells or fibroblast cells. Additionally, comparison of the list of methylated genes from another recent study [49] with our HKGs and TEGs did not yield any significant overlapping genes (data not shown). Based on these two pieces of evidence, HKGs do not appear to be enriched for DNA methylation, despite enrichment for CpG islands. This observation is consistent with previous reports that CpG islands in normal tissues are protected from methylation and that methylation of CpG islands is one of the mechanisms of tumorigenesis [50-52].

## Conclusion

Using high quality microarray gene expression profiling data, we identified a small subset of housekeeping genes that are highly expressed in 42 diverse normal tissues with small variation in expression level across these tissues. Cross species studies indicate that the functions and expression patterns of these HKGs are conserved between different species. These features make these genes better candidates for experimental references of transcription and expression levels than currently commonly used housekeeping genes: they can be easily detected, are stable across different tissues and are likely to be HKGs in other species. To investigate the mechanisms behind transcriptional regulation of HKGs and TEGs, we compared genomic features, chromatin structure, and epigenetic modifications between a larger set of HKGs and TEGs. We find that CpG islands are enriched near the TSSs of HKGs, in line with previous studies. HKGs have lower nucleosome occupancy, as indicated by strong enrichment of DNase I hypersensitive sites in HKGs that cannot be fully explained by the high expression level of HKGs in a single tissue type (CD4+T-cells). HKGs are enriched for DNase I hypersensitive sites relative to RefSeq genes of comparable or higher expression levels. HKGs and TEGs show significant differences in various histone methylation patterns, suggesting that histone methylation likely plays a role in the differential expression patterns but the relationships between histone methylation patterns and expression patterns is complex. DNA methylation patterns, in contrast, are similar for both HKGs and TEGs, suggesting that DNA methylation does not play a significant role in the differential expression patterns of these different types of genes. Elevated histone acetylation is not seen for HKGs after the correlation with expression is accounted for. Interestingly, however, histone acetylation appears to be elevated in all genes with moderate to high expression levels, suggesting that histone acetylation may serve as a general transcriptional switch to open chromatin and provide access to other transcription factors, which then regulate the extent of expression.

## Methods

### Tissues

mRNA from human tissues was purchased from commercial vendors, including Clontech, Ambion, and Biochain. Most samples were pooled from multiple donors, typically twelve.42 normal tissues were tested, in cluding adipose, adrenal gland, bladder, activated CD4-positive T-lymphocyte, activated CD8-positive T-lymphocyte, bone marrow, brain, fetal brain, cerebellum, cerebral cortex, hippocampus, thalamus, pituitary gland, cervix uteri, colon, epididymis, heart, kidney, fetal kidney, liver, fetal liver, lung, fetal lung, trachea, lymph node, mammary gland, skeletal muscle, ovary, placenta, prostate, retina, salivary gland, skin, duodenum, ileum, jejunum, spinal cord, spleen, stomach, testis, thymus, and thyroid gland. These selected tissues cover most major organs and normal tissue types. Four fetal tissues of brain, kidney, liver and lung were included.

### Microarray expression profiling

Human tissue microarray expression profiling was performed as described previously [53]. In brief, purchased mRNA pooled from multiple normal individuals was amplified and labeled using a full-length amplification protocol and hybridized in duplicate against a common reference pool in a two-color dye swap experiment [54]. Each gene is represented by 3 microarray probes placed at exon-exon junctions or in exons. Gene expression was calculated as the median probe intensity, after normalization by the pool of all data. The dataset is available at National Center for Biotechnology Information's Gene Expression Omnibus database [GEO accession: GSE16546].

### Selection of HKGs and TEGs

We used fairly conservative criteria to identify HKGs: the intensity of the gene must be greater than the median intensity of all genes in the microarray in at least 41 out of 42 tissues and the coefficient of variance (CV, standard deviation/average) of the gene intensity across tissues must be less than 1. The intensity and CV of the 18,149 genes monitored in the microarray are distributed over a wide range, with average intensity of all genes 1.04 ± 1.94 (SD) and average CV of all genes 0.83 ± 0.77 (SD). A recent study shows that genes' breadth of expression in tissues is positively correlated with the expression level of the genes [24]. Therefore it is reasonable to select HKGs from among those genes with higher intensity. While the CVs of most genes (76% of all genes) are below 1, some genes' expression is very volatile across tissues, with CV as high as 6. Our criteria guarantee the HKGs are highly expressed in vast majority of tissues with limited fluctuation in intensity level across tissues.

More stringent criteria were used to identify a reference HKG list for laboratory experimental controls. We required that the intensity of each HKG be greater than the median of all genes in each of the 42 tissues and CV of intensity less than 0.35. A total of 362 HKGs meet these criteria. The top 20 genes ranked by their average intensity across all 42 tissues were selected as the experimental housekeeping genes reference.

To identify TEGs, we selected 29 representative tissue types, removing fetal and redundant tissues from the set of 42 tissues described above. The resulting set was as follows: adipose, adrenal gland, bladder, bone marrow, brain, cervix uteri, colon, heart, kidney, liver, lung, trachea, mammary gland, ovary, skeletal muscle, lymph node, placenta, prostate, retina, salivary gland, skin, spinal cord, spleen, stomach, testis, thymus, thyroid gland, jejunum, and CD4-positive T-lymphocyte. To be identified as a TEG, the intensity of the gene in the relevant tissue was required to meet three criteria: 1) among the top 25% percentile of all genes in that particular tissue; 2) greater than 50% of the sum of intensities for that gene in all other tissues in the set of 29; and 3) greater than three times of intensity of the gene of interest in any other tissue.

### Conservation of functions

We used the number of orthologs of human genes in other eukaryotic species as identified by NCBI HomoloGene [23] as an indication of functional conservation across species. We mapped human HKGs, TEGs and all genes represented in the microarray to orthologs in mouse, rat, dog, fly (*D. melanogaster*), worm (*C. elegans*) and budding yeast (*S. cerevisiae*). The numbers of human genes that map to genes of other species through HomoloGene are counted. Student's T-tests were applied between orthologs of HKG and all genes and between orthologs of TEG and all genes.

### Distribution of SD and CNV in genes

We required at least a quarter of the total genomic length of a gene to overlap the SD or CNV region (Table 3). The p-values, indicating the statistical significance of the overlap for HKGs and TEGs relative to all RefSeq genes, were calculated according to the hypergeometric distribution with a Bonferroni correction.

### CpG islands

CpG islands coordinates were obtained from UCSC genome browser  human CpG island track. The number and length of CpG islands located within 500 bp upstream and downstream of transcription start sites and end sites are calculated for HKGs, TEGs and RefSeq genes. CpG density is indicated by the fraction of base pairs occupied by CpG islands. The hypergeometric distribution with Bonferroni correction is applied to determine the significance of the enrichment or depletion of CpG islands relative to the density seen for RefSeq genes.

### Chromatin structure and epigenetics modifications

Data of DNase I hypersensitive (HS) sites, histone acetylation, methylation, transcription binding sites and DNA methylation were obtained from recent publications [35,39,43,48]. The density of each feature is calculated in a 500 bp sliding window advancing 100 bp each time near transcription start sites for HKGs, RefSeq genes, TEGs. The average intensity of all genes in each group is plotted as a function of the distance to transcription start site.

## Authors' contributions

XS carried out the analysis. XS and RC designed the analysis. CR and JJ participated in discussions and provided valuable suggestions. JC and AK carried out microarray probe design and intensity analysis. JJ conceived of the study. XS, CR and RC wrote the manuscript. All authors helped to draft the manuscript and approved the final manuscript.

## Supplementary Material

Additional file 1 **Housekeeping gene list**. List of 1552 human housekeeping genes. Click here for file

Additional file 2 **Tissue enriched gene list**. List of 975 human tissue enriched genes. Click here for file

Additional file 3 **Comparison of housekeeping genes identified in different studies**. Venn diagram of housekeeping genes identified in three different studies. Click here for file

Additional file 4 **Comparison of tissue enriched/specific genes identified in different studies**. Venn diagram of tissue enriched genes of four tissues identified in two different studies. Click here for file

Additional file 5 **Biological processes of tissue-enriched genes**. A list of enriched biological processes of tissue-enriched genes. Click here for file

Additional file 6 **DNA methylation at transcription start sites**. Genome wide DNA methylation level around transcription start sites in sperm and fibroblast cell lines. Click here for file
