# Supplementary material for: Definition, conservation and epigenetics of housekeeping and tissue-enriched genes
Source: BMC Genomics. 2009 Jun 17;10:269. doi: 10.1186/1471-2164-10-269 (PMC2706266; doi:10.1186/1471-2164-10-269)
Supplement: Additional file 3 — Comparison of housekeeping genes identified in different studies. Venn diagram of housekeeping genes identified in three different studies. [file 1471-2164-10-269-S3.pdf]

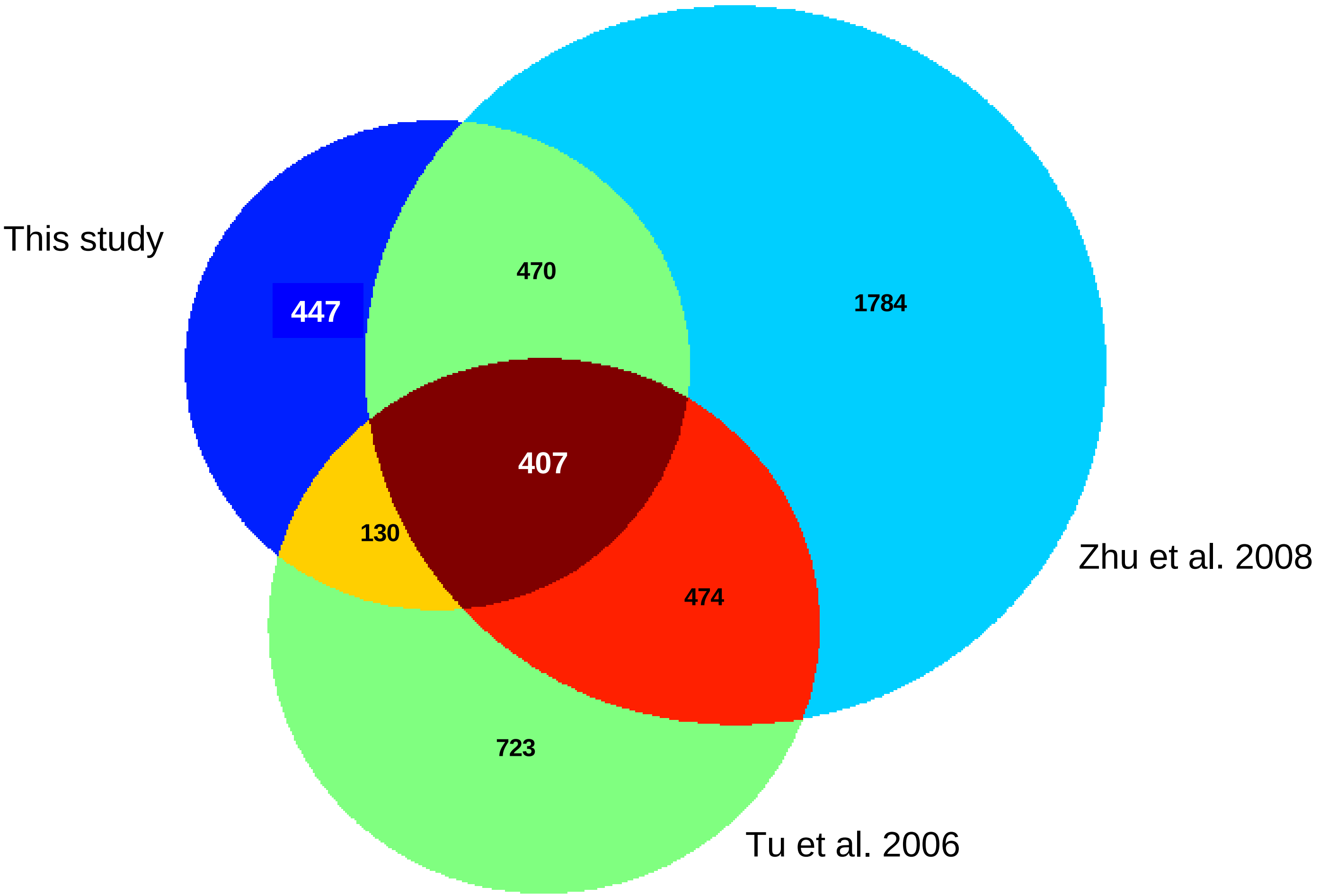

Additional file 3. Comparison of housekeeping genes identified in different studies

The numbers are the housekeeping genes identified by each study alone or those common between any two or all studies. Genes of this study and Tu et al. are based on microarray dataset, while genes of Zhu et al. are based on EST dataset. The Venn diagram is drawn to the scale of the numbers. See main text for reference.
