# Supplementary material for: Definition, conservation and epigenetics of housekeeping and tissue-enriched genes
Source: BMC Genomics. 2009 Jun 17;10:269. doi: 10.1186/1471-2164-10-269 (PMC2706266; doi:10.1186/1471-2164-10-269)
Supplement: Additional file 4 — Comparison of tissue enriched/specific genes identified in different studies. Venn diagram of tissue enriched genes of four tissues identified in two different studies. [file 1471-2164-10-269-S4.pdf]

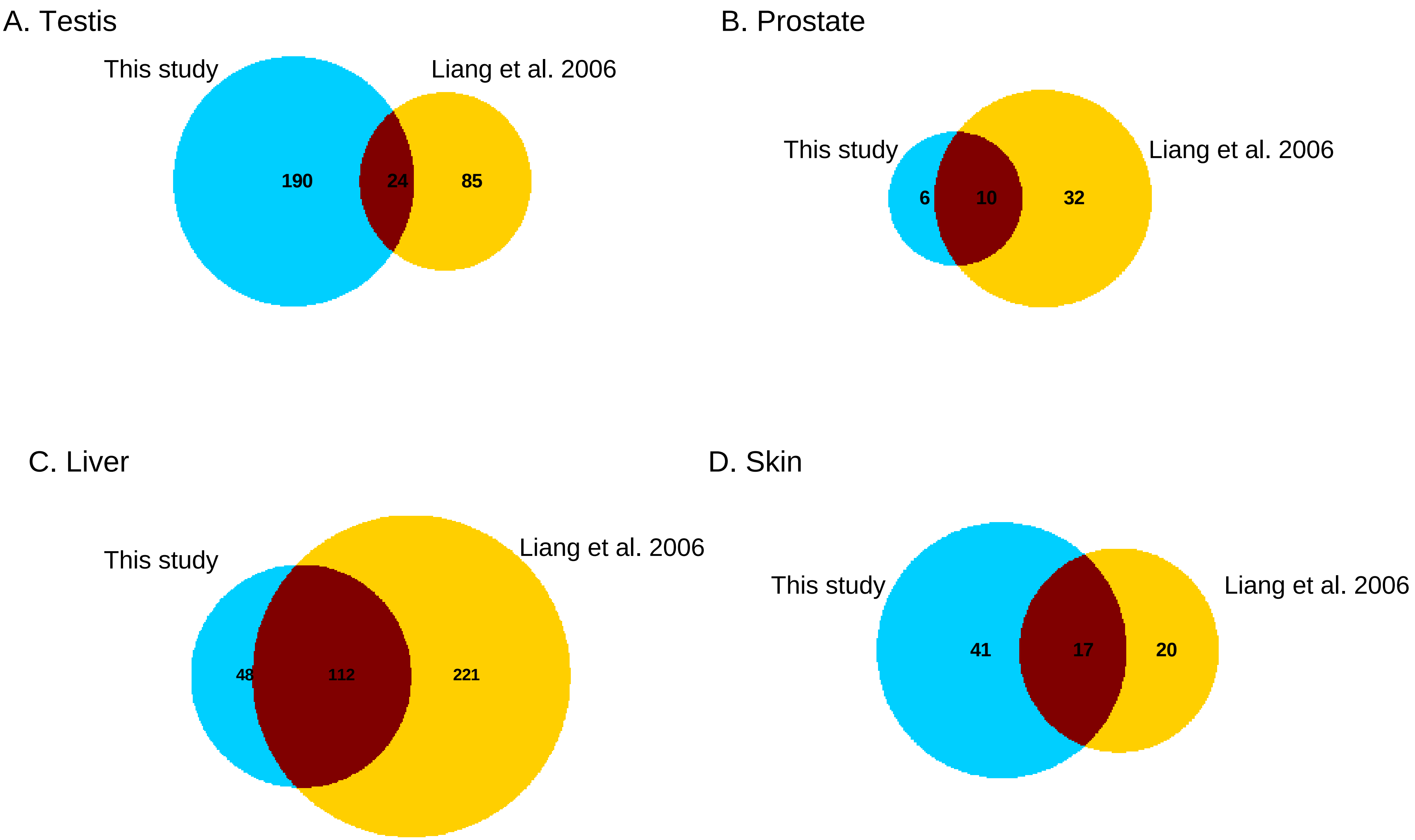

Additional file 4. Comparison of tissue enriched/specific genes identified in two studies The numbers are the tissue enhanced/specific genes in testis (A), prostate (B), liver (C) and skin (D) identified by each study alone or those common between the two studies. See main text for reference.
