# Supplementary material for: Definition, conservation and epigenetics of housekeeping and tissue-enriched genes
Source: BMC Genomics. 2009 Jun 17;10:269. doi: 10.1186/1471-2164-10-269 (PMC2706266; doi:10.1186/1471-2164-10-269)
Supplement: Additional file 6 — DNA methylation at transcription start sites. Genome wide DNA methylation level around transcription start sites in sperm and fibroblast cell lines. [file 1471-2164-10-269-S6.pdf]

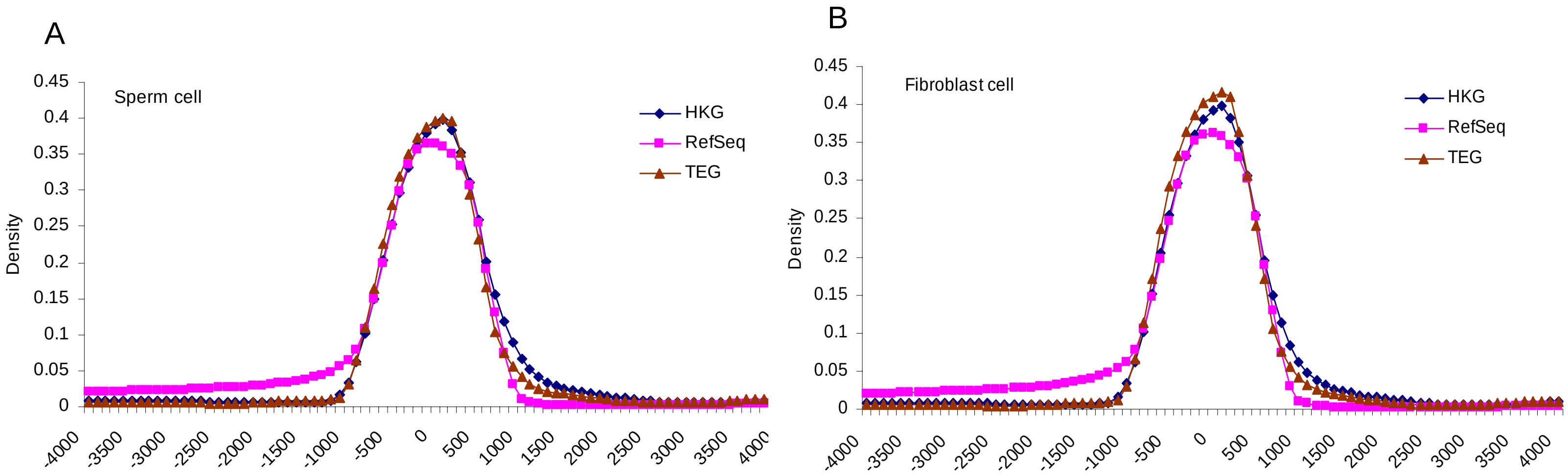

Additional file 6. DNA methylation at transcription start sites.

DNA methylation was detected in sperm cells (A) and fibroblast cells (B) (Weber et al. 2007). The average density of DNA methylation in three gene groups, HKGs, RefSeq genes and TEGs, are calculated in each 500 bp window 4 kb around the transcription start sites.
